# Obstructive sleep apnea in young Asian adults with sleep-related complaints

**DOI:** 10.1038/s41598-022-25183-5

**Published:** 2022-11-29

**Authors:** Hwa-Yen Chiu, Kun-Ta Chou, Kang-Cheng Su, Fang-Chi Lin, Yung-Yang Liu, Tsu-Hui Shiao, Yuh-Min Chen

**Affiliations:** 1 Center of Sleep Medicine, Taipei Veterans General Hospital, 14F, No. 201, Sec. 2, Shih-Pai Road, Taipei, 112 Taiwan; 2 Division of Clinical Respiratory Physiology, Department of Chest Medicine, Taipei Veterans General Hospital, Taipei, Taiwan; 3 Department of Internal Medicine, Taipei Veterans General Hospital Hsinchu Branch, Zhudong, Hsinchu County Taiwan; 4 Faculty of Medicine, School of Medicine, National Yang Ming Chiao Tung University, Taipei, Taiwan; 5 Institute of Biophotonics, National Yang Ming Chiao Tung University, Taipei, Taiwan

**Keywords:** Epidemiology, Ageing, Respiration

## Abstract

This study aimed to investigate the proportion of young OSA adults with sleep-related complaints in a sleep center, affiliated with a tertiary medical center for over a decade. This study presents a chronicle change in the numbers of young adults receiving polysomnography (PSG) and young patients with OSA from 2000 to 2017. We further analyzed 371 young patients with OSA among 2378 patients receiving PSG in our sleep center from 2016 to 2017 to capture their characteristics. Young adults constituted a substantial and relatively steady portion of examinees of PSG (25.1% ± 2.8%) and confirmed OSA cases (19.8 ± 2.4%) even though the total numbers increased with the years. Young adults with OSA tend to be sleepier, have a greater body mass index, and have a higher percentage of cigarette smoking and alcohol consumption. They also complained more about snoring and daytime sleepiness. They had a higher apnea–hypopnea index on average and experienced more hypoxemia during their sleep, both in terms of duration and the extent of desaturation. Even though the prevalence of comorbidities increased with age, hypertension in young male adults carried higher risks for OSA. Young adults with OSA have constituted a relatively constant portion of all confirmed OSA cases across time. The young adults with OSA were heavier, more symptomatic, and with more severe severity.

Clinical trial: The Institutional Review Board of Taipei Veterans General Hospital approved the study (VGHIRB No. 2018-10-002CC). The study is registered with ClinicalTrials.gov, number NCT03885440.

## Introduction

Obstructive sleep apnea (OSA) is a common sleep-related disorder characterized by upper airway obstruction during sleep, which results in breathing pause, intermittent desaturation, and frequent arousals. OSA is defined as apnea–hypopnea index (AHI) ≥ 5 times/hour with a prevalence of 10% in the US^1^. In Taiwan, the prevalence of witnessed apnea was 2.6%, and the prevalence of snoring was up to 60%^2^. The prevalence of OSA might be even higher currently^3^. These patients were at risk or linked to a variety of diseases, such as hypertension^4^, cardiovascular disease^5^, insulin resistance^6^, and cognitive impairment^7^. Undiagnosed OSA was also linked to accelerated aging^8^.

OSA is an age-dependent disease. With increasing age, the prevalence of sleep apnea increases and reaches a plateau around 65 years old^9^. Ageing is considered one of the risk factors for OSA^10^, and is also included in both STOP-BANG^11^ and OSA50^12^ questionnaires. To explore the aging effects on OSA, most studies focused on middle-aged adults^13^ and elderlies^8,14,15^ with population-based approaches by either post survey^16^ or telephone review^9^. The association between OSA and the incidence of excessive daytime sleep^16^, hypertension^17^, and cardiovascular diseases^18^ declines in elderlies compared with middle-age adults^8^. Young adults aged less than 40 are usually neglected, even for large prospective cohort such as the Sleep Heart Health Study^19^. Considering the aging accelerating effect of OSA and the young adulthood is the prior stage before the middle-aged adulthood, the characteristics of young adults with OSA deserves to be clarified. Furthermore, the young adults with OSA were documented to utilize the healthcare system more than healthy controls^20^. The characteristics of symptomatic patients may be different from undiagnosed OSA in the general population. Hence, we conducted a retrospective study to investigate the proportion and the characteristics of young Asian adults with OSA who came to seek medical help with sleep-related complaints.

## Methods

### Design, setting and patients

This retrospective observational study was conducted in the Center of Sleep Medicine in Taipei Veterans General Hospital, Taipei, Taiwan, the first sleep Lab was founded in Taiwan in 1983^21^, where all the polysomnography (PSG) studies were performed by the Taiwan Society of Sleep Medicine (TSSM) board certificated sleep technicians. The whole night PSG reports from 2000 to 2017 were used to calculate the number and percentage of young adults in patients who received PSG for sleep problems.

Furthermore, we enrolled treatment naïve adults who received PSG in our sleep center between Jan 1, 2016, and December 31, 2017, to compare the characteristics in different age strata. All records of consecutive patients who received polysomnography were retrospectively reviewed. Patients aged less than 20 years or with incomplete sleep questionnaires were excluded. Enrolled patients were divided into 3 groups as different stages of adulthood^22^ for further analyses, including the Young adult group^22–24^ (20–40 years old), the Middle-aged adult group^25^ (41–60 years old), and the Older adult group^26^ (> 60 years old).

### Measurements

Baseline information including age, gender, weight, body mass index (BMI), and self-reported habits and medical history, Epworth’s sleepiness scale (ESS) were routinely obtained and recorded in our sleep center before polysomnography. Parameters in nocturnal PSG were recorded.

### Polysomnography

Overnight PSGs were performed using Alice 3–6 Diagnostics System during 2000–2017 ([Philips] Respironics Inc., USA) and Embla n7000 during 2015/10-2017 (< 10% cases, Embla Systems, Inc., Broomfield, Colorado, USA). PSGs include recordings of Electroencephalography (EEG), electrooculography (EOG), electromyography (EMG), chest and abdominal respiratory movements, nasal pressure cannulas, airflow monitor, and finger oxygen saturation.

Based on the criteria of the American Academy of Sleep Medicine (AASM) manual for the scoring of sleep and associated events, we scored every 30 s epoch of PSG. Apnea was defined as the complete cessation of airflow (≥ 90%) for at least 10 s. Hypopnea was constantly defined by airflow reduction lasting over 10 s with blood oxygen desaturation ≥ 4% throughout 2000–2017 despite that the extent of airflow reduction had ever been revised (≥ 30% from 2007 to 2017^27^, a discernible reduction from 2000 to 2007^28^)^29^. Apnea–hypopnea index (AHI) was defined as the total number of apneas and hypopneas per hour of sleep, and the oxygen desaturation index (ODI) was defined as the number of oxygen desaturations (≥ 4%) per hour of sleep. The severity of OSA was classified as mild (AHI ≥ 5 and < 15), moderate (AHI ≥ 15 and < 30) or severe (AHI ≥ 30)^30^.

### Statistical analysis

SPSS software (version 22.0; SPSS Inc., Chicago, IL) was used for all analyses. All continuous variables are presented as median (interquartile range) while they were non-normally distributed, and were compared using the Kruskal Wallis test. Pearson’s chi-square test was performed to compare categorical variables. Cochran–Armitage test was used for trend analysis. Sensitivity analysis was performed to evaluate desaturation duration. The incidence of OSA-related comorbidities was analyzed by Pearson’s chi-square test. Gender stratified analysis was used for patients with OSA to eliminate the gender effect. Univariate and multivariate logistic regression were used to evaluate the impact of the comorbidities on the prevalence of OSA. A two-sided p-value < 0.05 was considered statistically significant and a two-sided p < 0.017 were used in post hoc analysis for comparison between the three groups.

### Ethics

This study is approved by the Institutional Review Board, Taipei Veterans General Hospital (2018-10-002CC), and registered with ClinicalTrials.gov, number NCT03885440. This research was performed in accordance with relevant guidelines/regulations. Informed consent was waived due to its retrospective nature by the Institutional Review Board, Taipei Veterans General Hospital (2018–10-002CC). This research was performed in accordance with the Declaration of Helsinki.

## Results

**Figure 1:**
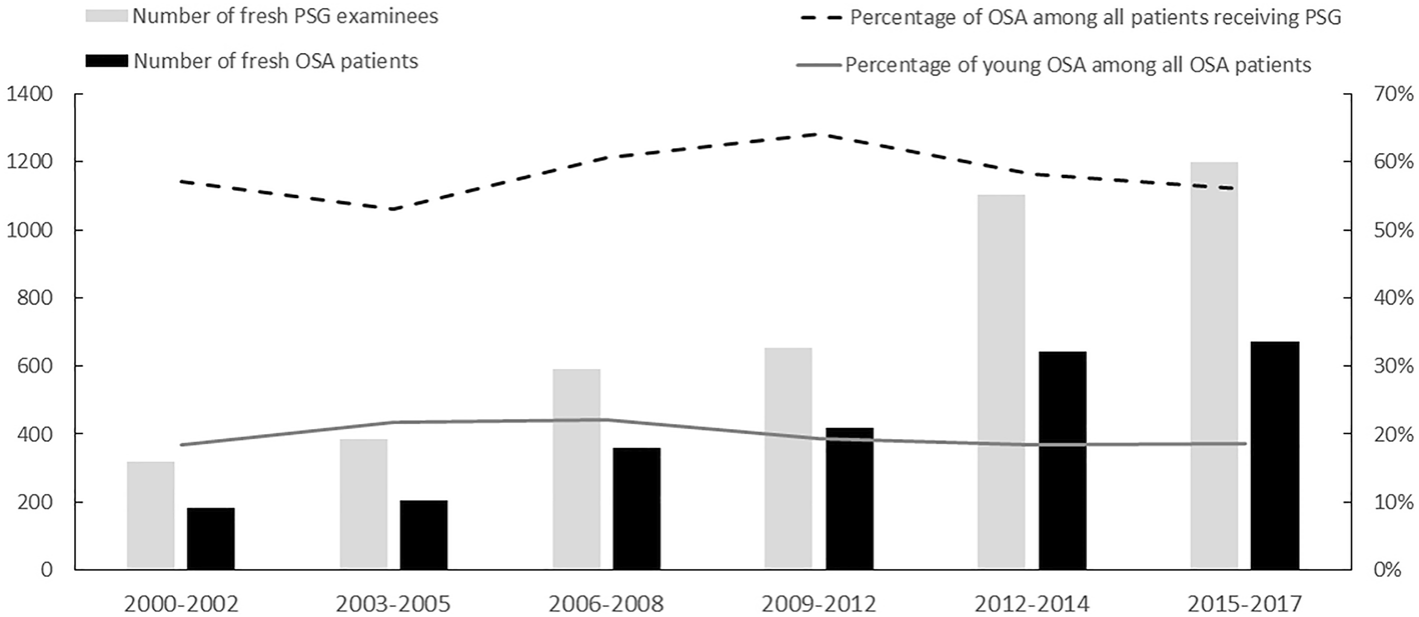
Young patients with OSA constituted a substantial portion of all patients with OSA, which did not vary significantly year to year (p value for trend = 0.162). The diagnosis of OSA was defined as apnea–hypopnea index ≥ 5 while hypopnea was defined as a discernible reduction in airflow accompanied by a decrease in oxyhemoglobin saturation of 4% before 2007 and a 30% decrease in nasal pressure with desaturation > 4% after 2007.

From 2000 to 2017, the number of young adults who received PSG increased year by year, accounting for 20.9–29.6% (25.1% ± 2.8%) of all the examinees. Among them, 53.1–64.1% were diagnosed with OSA (Fig. 1). The confirmed younger patients with OSA accounted for 19.8% ± 2.4% of all confirmed cases. The trends of mean BMI of young adults who received PSG and those with OSA were shown in Fig. S1. There was a trend of increasing BMI in young adults with OSA but not in all young adult examinees.

**Figure 2:**
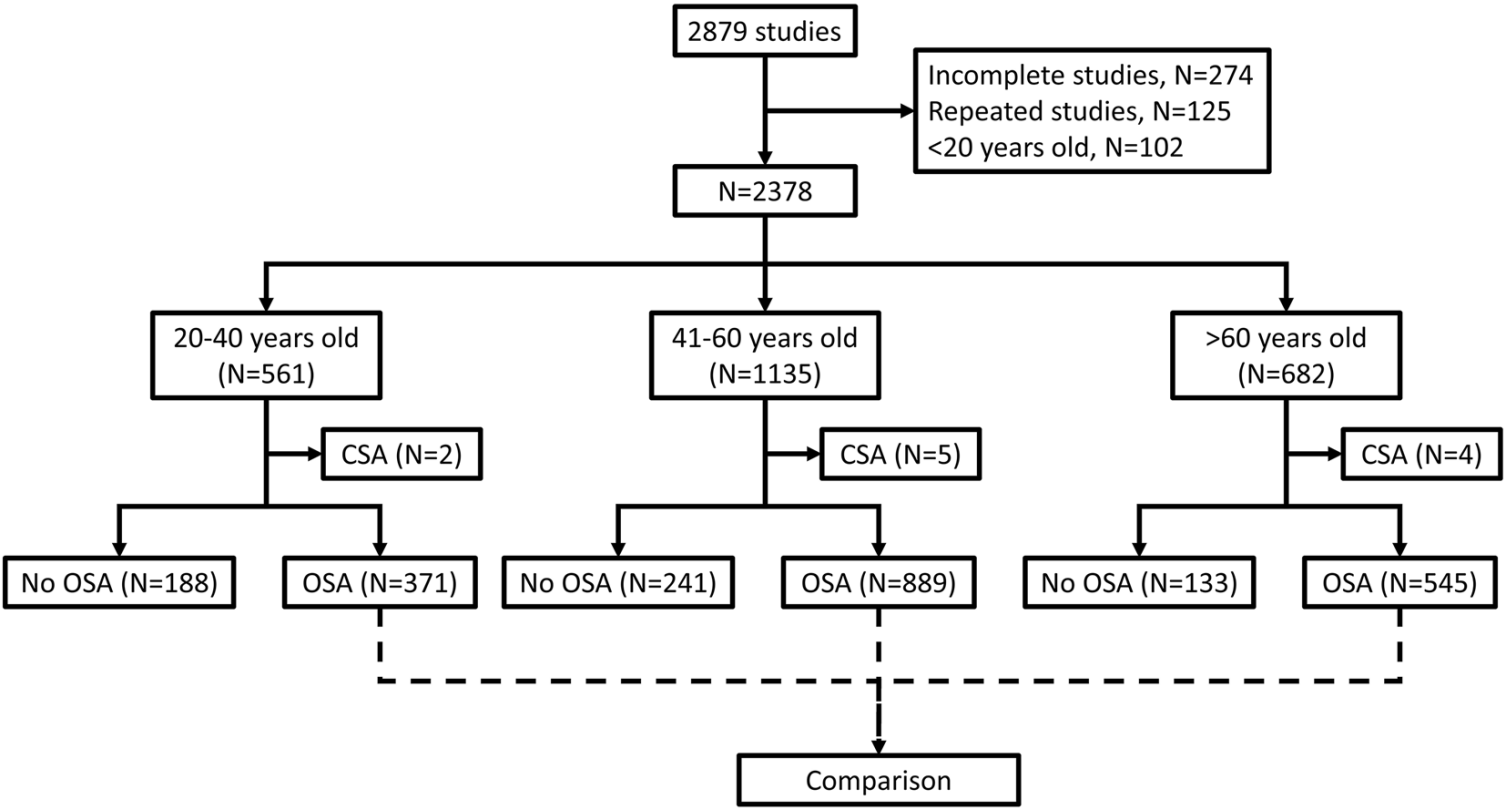
The flow diagram of comparison of patients with OSA across age stratums.

From Jan 1st, 2016 to Dec 31st, 2017, a total of 2879 polysomnography studies were performed in the Center of Sleep Medicine in Taipei Veterans General Hospital, Taipei, Taiwan. After excluding 274 incomplete studies, 125 follow-up studies, and 102 studies with participants less than 20 years old, 2378 studies went into the final analysis (Fig. 2). 371 of 561 young adults (20–40 years old), 889 of 1135 middle-aged adults, and 545 of 682 older adults (> 60 years old) were diagnosed with OSA.

As shown in Table 1, in patients receiving PSG due to sleep problems, the proportion of males is greater in the young adult group. The young adults are slightly heavier and have more complaints about snoring and daytime sleepiness. On the contrary, a greater proportion of older adults experienced dozing off while watching TV. A larger proportion of young adults and middle-aged adults reported dozing off while driving than older adults. The prevalence of OSA increased with age. The proportion of patients with central sleep apnea showed no significant difference between the three groups.

**Table 1:** Characteristics of enrollees.

Age (years)	20–40	41–60	> 60	p value
N	561	1135	682	
Age, years old, median	33	52	67	
Male, N (%)	457 (81.5)*^+^	**840 (74)**	**478 (70.1)**	** < 0.001**
BMI, kg/m^2^, median (IQR)	**27.2 (23.7–31.6)**^**+**^	**26.6 (24.1–29.4)**^**#**^	**25.7 (23.5–28.6)**	** < 0.001**
ESS, median (IQR)	7 (4–9.5)*^+^	6 (4–9)	5 (3–8)	< 0.001
CSA, N (%)	2 (0.4)	5 (0.4)	4 (0.6)	0.828
OSA, N (%)	371 (66.1)*^+^	889 (78.3)	545 (79.9)	< 0.001
**Chief complaint**				
Snoring	506 (90.2)^+^	1008 (88.8)^#^	563 (82.6)	< 0.001
Daytime sleepiness	503 (89.7)*^+^	939 (82.7)^#^	515 (75.5)	< 0.001
Dozing off while watching TV	264 (47.1)*^+^	621 (54.7)^#^	408 (59.8)	< 0.001
Dozing off while driving	194 (34.6)^+^	415 (36.6)^#^	106 (15.5)	< 0.001
**Habits**				
Smoking	171 (30.5)^+^	358 (31.5)^#^	150 (22.0)	< 0.001
Drinking	339 (60.4)*^+^	576 (50.7)^#^	279 (40.9)	< 0.001
**Comorbidities**				
Hypertension, N (%)	94 (16.8)*^+^	389 (34.3)^#^	359 (52.6)	< 0.001
Heart disease, N (%)	11 (2.0)*^+^	101 (8.9)^#^	169 (24.8)	< 0.001
Stroke, N (%)	3 (0.5)*^+^	34 (3.0)^#^	57 (8.4)	< 0.001
Diabetes mellitus, N (%)	21 (3.7)*^+^	119 (10.5)^#^	127 (18.6)	< 0.001
Hyperlipidemia, N (%)	96 (17.1)*^+^	403 (35.5)	238 (34.9)	< 0.001
Anxiety, N (%)	73 (13.0)^+^	193 (17.0)^#^	151 (22.1)	< 0.001
Depression, N (%)	33 (5.9)^+^	93 (8.2)	72 (10.6)	0.012

*BMI* body mass index, *CSA* central sleep apnea, *ESS* Epworth sleepiness score, *IQR* interquartile range, *OSA* obstructive sleep apnea.

Significant values are given in bold.

*Post-hoc p < 0.017 young adults (20–40 years) v.s. middle age adults (40–60 years).

^+^Post-hoc p < 0.017 young adults v.s. older adults (> 60 years).

^#^Post-hoc p < 0.017 middle age adults v.s. elder adults.

The characteristics of patients with OSA are shown in Table 2 and Table S1 in the supplemental material. The BMI was greater in young adult groups of both gender, and young men with OSA were found sleepier and with more arousals than older men with OSA. The sleep efficiency decreased with age in both genders. Though the severity of OSA didn’t show a significant difference between the 3 groups, a trend showed that young men were more desaturated than middle-aged men and older men. SpO2 nadir and desaturation duration were worse in young men with OSA. The longer desaturation duration in young men was also confirmed via sensitivity analysis. Among 4 recorded sleep-related complaints, the characteristics varied depending on age and sex. The prevalence of sleep-related comorbidities, such as hypertension, heart disease, stroke, and hyperlipidemia increased with age. The prevalence of diabetes and anxiety increased with age in men but not in women. The prevalence of depression did not make a significant difference due to aging in both genders.

**Table 2:** Characteristics of patients with OSA.

	20–40 year-old	41–60 year-old	> 60 years old	p value
N	371	889	545	
Age, years old, median	34 (29–38)	53 (48–56)	67 (64–73)	
Male, N (%)	340 (91.6)*^+^	714 (80.3)^#^	398 (73.0)	< 0.001
BMI, kg/m^2^, median (IQR)	29.4 (26.0–33.2)*^+^	27.3 (24.9–30.3)^#^	26.2 (23.9–29.1)	< 0.001
BMI < 24, N (%)	47 (12.7)*^+^	152 (17.1)^#^	141 (25.9)	
24 ≤ BMI < 27, N (%)	75 (20.2)	259 (29.1)	168 (30.8)	
BMI ≥ 27, N (%)	249 (67.1)	478 (53.8)	236 (43.3)	
ESS, median (IQR)	7 (4–10)^+^	6 (4–9)	6 (3–8)	< 0.001
ESS > 10, N (%)	67 (18.1)^+^	14 (16.6)	78 (14.3)	< 0.001
AHI, median (IQR)	31.1 (14.1–62.3)*^+^	**26.8 (12.8–51.4)**	**26.3 (13.8–48.4)**	< 0.001
**OSA grade**				
Mild, N (%)	101 (27.2)	275 (30.9)	159 (29.2)	0.475
Moderate, N (%)	83 (22.4)	210 (23.6)	137 (25.1)	
Severe, N (%)	187 (50.4)	404 (45.4)	249 (45.7)	
RERA index, median (IQR)	8.6 (4.2–14.3)	8.7 (4.8–14.8)	8.8 (4.2–15.6)	0.845
Sleep efficiency, %, median (IQR)	85.1 (71.9–91.5) *^+^	82.1 (72.5–88.2)^#^	75.0 (63.0–83.8)	< 0.001
Arousal index, median (IQR)	16.1 (10.4–27.5)*^+^	**14.2 (8.7–24.1)**^#^	**13.2 (7.9–20.6)**	< 0.001
ODI, median (IQR)	28.6 (12.2–57.4)*^+^	23.3 (10.8–46.3)	23.0 (10.7–44.8)	0.006
SpO_2_ nadir, %, median (IQR)	79 (70–84)*^+^	80 (73–85)	81 (75–86)	< 0.001
SpO_2_ < 90%, sec, median (IQR)	10.3 (2.1–44.3)^+^	8.7 (1.9–31.6)	7.5 (2.0–28.0)	0.040
SpO_2_ < 85%, sec, median (IQR)	1.5 (0–12.3)^+^	1.0 (0–6.7)^#^	0.5 (0–4.9)	< 0.001
SpO_2_ < 80%, sec, median (IQR)	0.1 (0–2.0)^+^	0 (0–1.4)	0 (0–0.7)	< 0.001
**Chief complaint**				
Snoring	350 (94.3)*^+^	787 (88.5)^#^	455 (83.5)	< 0.001
Daytime sleepiness	333 (89.8)*^+^	722 (81.2)^#^	409 (75.0)	< 0.001
Dozing off while watching TV	191 (51.5)^+^	502 (56.5)	330 (60.6)	0.024
Dozing off while driving	140 (37.7)^+^	350 (39.4)^#^	87 (16.0)	< 0.001
**Habits**				
Smoking	131 (35.3)^+^	298 (33.5)^#^	121 (22.2)	< 0.001
Drinking	238 (64.2)*^+^	458 (51.5)^#^	228 (41.8)	< 0.001
**Comorbidities**				
Hypertension, N (%)	81 (21.8)*^+^	330 (37.1)^#^	296 (54.3)	< 0.001
Heart disease, N (%)	8 (2.2)*^+^	79 (8.9)^#^	134 (24.6)	< 0.001
Stroke, N (%)	3 (0.8)*^+^	28 (3.1)^#^	46 (8.4)	< 0.001
Diabetes mellitus, N (%)	20 (5.4)*^+^	98 (11.0)^#^	106 (19.4)	< 0.001
Hyperlipidemia, N (%)	76 (20.5)*^+^	337 (37.9)	189 (34.7)	< 0.001
Anxiety, N (%)	45 (12.1)^+^	130 (14.6)^#^	115 (21.1)	< 0.001
Depression, N (%)	17 (4.6)^+^	66 (7.4)	56 (10.3)	0.006

*AHI* apnea–hypopnea index, *BMI* body mass index, *CSA* central sleep apnea, *ESS* Epworth sleepiness score, *IQR* interquartile range, *ODI* oxygen desaturation index, *RERA* respiratory effort related arousal.

Significant values are given in bold.

*Post-hoc p < 0.017 young adults (20–40 years) v.s. middle age adults (40–60 years).

^+^Post-hoc p < 0.017 young adults v.s. older adults (> 60 years).

^#^Post-hoc p < 0.017 middle age adults v.s. elder adults.

A univariate logistic regression showed that gender and obesity were associated with OSA (Table 3). Gender stratified analyses in Table S2 in the supplemental material showed that the odds ratios (ORs) of obesity, hypertension, and hyperlipidemia diminished from the younger to the older groups. Multivariate logistic regression analyses were done to explore the association between patient characteristics and OSA among young adults in Table 4. Patients with OSA had an odds ratio of obesity at 6.05 (3.67–9.96) in men and 6.28 (2.10–18.76) in women. For young men with OSA, the odds ratio of hypertension was 2.50 after adjustment for obesity. For women with OSA, the risk for diabetes mellitus and hyperlipidemia did not reach statistical significance.

**Table 3:** Uni-variate logistic regression for odds ratio of characteristics related prevalence changes of OSA according to age.

	20–40 year-old	41–60 year-old	> 60 years old
Male	6.00 (3.47–10.39)**	3.01 (2.11–4.28)**	2.20 (1.39–3.48)**
Smoking	1.28 (0.78–2.08)	0.97 (0.66–1.42)	0.68 (0.41–1.16)
Drinking	1.18 (0.76–1.85)	0.87 (0.62–1.20)	0.98 (0.63–1.53)
Obesity	6.07 (3.81–9.68)**	4.15 (2.99–5.76)**	2.22 (1.48–3.35)**
Hypertension	1.73 (0.85–3.53)	1.46 (1.00–2.10)	1.23 (0.81–1.88)
Heart disease	1.88 (0.26–13.66)	0.63 (0.35–1.11)	0.90 (0.56–1.46)
Stroke	^$^	1.49 (0.54–4.06)	1.07 (0.52–2.19)
Diabetes mellitus	8.46 (0.98–72.79)	0.99 (0.56–1.75)	1.21 (0.69–2.11)
Hyperlipidemia	0.98 (0.52–1.85)	1.30 (0.91–1.85)	1.02 (0.66–1.57)
Anxiety	1.23 (0.59–2.56)	0.75 (0.49–1.16)	0.82 (0.50–1.37)
Depression	0.46 (0.16–1.29)	0.85 (0.47–1.53)	1.03 (0.52–2.05)

Significant values are given in bold.

^$^Not available, *p < 0.05, **p < 0.01.

**Table 4:** Independent factors associated with OSA among young adults.

Male	Odds ratio	p value	Female	Odds ratio	p value
Obesity	**6.05 (3.67–9.96)**	< 0.001	Obesity	**6.28 (2.10–18.76)**	0.001
Hypertension	**2.50 (1.09–5.75)**	0.031	Diabetes mellitus	2.94 (0.27–32.71)	0.380
			Hyperlipidemia	4.30 (0.84–21.92)	0.079

Significant values are given in bold.

## Discussion

This study showed young adults accounted for a substantial and steady portion (19.8% ± 2.4%) of patients with OSA from 2000 to 2017. Young adults with OSA were more obese, sleepier, and had a higher percentage of alcohol consumption compared with counterparts in the other two age stratum. Despite fewer comorbid diseases among young adults with OSA, hypertension confers a higher risk of OSA for young adults, especially for males, which is compatible with the study by Asha'ari ZA^31^. Additionally, we also found that > 70% (72.8%) of the young adults with OSA are of moderate to severe severity, for whom aggressive treatment is generally recommended.

While age > 40 years is an independent risk factor for OSA by US Preventive Services Task Force^10^, past studies mainly enrolled middle-aged adults and elderlies to evaluate the incidence of OSA^13,28,32,33^, the pathophysiology^9^ and the comorbidities^14,34,35^. Some studies also discussed OSA in childhood or adolescents, highlighting that children are not little adults^36^. In contrast, young adults aged from 20 to 40 are often neglected, which is a discrete category.

In our study, young adults with OSA are more obese, similar to OSA in adolescents^37^. We further demonstrated that young adults with OSA are sleepier and more desaturated. This finding accorded with previous research showing that AHI, ODI, and desaturation severity are related to excessive daytime sleepiness^38^, which would impair performance and diminish the quality of life. In our study, young adults with sleep complaints and/or OSA had a higher percentage of alcohol consumption. Though alcohol drinking is not a risk factor for OSA in the current study, its implication on OSA in young adults awaits further exploration.

The pathophysiology of OSA in young adults may be contributed by obesity and anatomical limitation as adolescents. As a 1% change in body weight was associated with a 3% change in AHI in middle-aged adults^39^, our study showed obesity is the strongest risk factor of OSA in young adults. Moreover, the increasing obesity prevalence in the recent decade^40^, particularly for Asians, would hence contribute to the risk of OSA for young adults. The temporal analysis part of our study also demonstrated that the BMI of young adults with OSA increased year to year from 2000 to 2017 (Fig. S1). The upper airway anatomical trait of young adults may contribute to the emergence of OSA. While tonsillar hypertrophy has been shown associated with childhood OSA and OSA in adults^41^, young adults with tonsillar hypertrophy were reported to be at higher risk for OSA^31^.

In this study, we also evaluated the sleepiness of our patients. More severe daytime sleepiness could drive young adults to the sleep center with a positive effect on the detection rate. However, there was only a small difference in ESS scores (7 vs. 6) between young adults and the other two age groups while the minimal clinically important difference in ESS scores is 2–3^42,43^. Apart from symptoms, comorbidities are another factor that affects the will of seeking medical help. After adjusting for obesity, we found the only comorbidity associated with OSA is hypertension in males, which is similar to the previous study^31^.

The strength of our study is that we collected reports with relatedly consistent scoring criteria from the earliest sleep center in Taiwan^21^. All the technicians are certified by TSSM and audited bimonthly to assure inter-scorer reliability. Therefore, we could provide reliable data about epidemiology on a larger temporal scale. Furthermore, the patients were routinely surveyed for symptoms (ESS, etc.) and comorbidities with questionnaires with the help of technicians before each polysomnography; hence, the comorbidities are more accurate in our study than by chart review only.

Nevertheless, this study is still limited by its retrospective nature. Moreover, the study participants were recruited from a sleep center affiliated with a tertiary medical center, probably consisting of participants presenting with more symptoms or comorbid diseases. A detection bias would lead to an overestimated impact of OSA despite fewer comorbid diseases reported among young adults in this study. Furthermore, we had ever revised criterion of hypopnea in 2007 regarding the extent of airflow reduction (≥ 30% from 2007 to 2017^27^; a discernible reduction from 2000 to 2007^28^), thereby raising the concern of consistency in evaluating the chronicle change in prevalence of OSA among young adults. It is noteworthy that the recommended definition of hypopnea shifted with time during the late 1990s to early 2000s. In this study, hypopnea was constantly defined by airflow reduction lasting over 10 s with oxyhemoglobin desaturation ≥ 4% from 2000 to 2017. Compared with a 3% drop in oxyhemoglobin desaturation, the higher cut-off (≥ 4%) could provide more stable documentation for respiratory events.

## Conclusion

Young patients with OSA have constituted a substantial portion of all patients with OSA and > 70% of them are of moderate to severe severity. Clinicians are advised to keep alert for young adults with suspected traits and initiate work-up for early detection of OSA, especially for those with obesity or hypertension.

## Supplementary Information

Supplementary Information.

## Data Availability

The datasets generated during and/or analyzed during the current study are available from the corresponding author on reasonable request.

## Abbreviations

AHI: Apnea–hypopnea index
BMI: Body mass index
EEG: Electroencephalography
ESS: Epworth’s sleepiness scale
IODI: Oxygen desaturation index
OSA: Obstructive sleep apnea
PSG: Polysomnography
TSSM: Taiwan Society of Sleep Medicine
