# Supplementary material for: Obstructive sleep apnea in young Asian adults with sleep-related complaints
Source: Sci Rep. 2022 Nov 29;12:20582. doi: 10.1038/s41598-022-25183-5 (PMC9708676; doi:10.1038/s41598-022-25183-5)
Supplement: Supplementary file 1 — Supplementary Information. [file 41598_2022_25183_MOESM1_ESM.docx]

**Supplemental Materials:**

Table S1 Characteristics of patients with OSA stratified according to sex and age

|  | | Male | | | | Female | | | |
| --- | --- | --- | --- | --- | --- | --- | --- | --- | --- |
|  | 20~40 year-old | | 41~60 year-old | >60 years old | p value | 20~40 year-old | 41~60 year-old | >60 years old | p value |
| N | 340 | | 714 | 398 | 1452 | 31 | 175 | 147 | 353 |
| Age, years old, median | 34 | | 52 | 67 |  | 35 | 54 | 68 |  |
| BMI, kg/m^2^, median(IQR) | **29.3(26.1-33) ^* +^** | | **27.4(25.1-30.4)^#^** | **26.2(24.1-28.9)** | **.000** | **30.5(25.9-35.4) ^* +^** | **26.6(23.4-29.6)** | **26.1(23.4-30.6)** | **.020** |
| BMI<24, N(%) | 42(12.4) ^* +^ | | 100(14.0)^#^ | 95(23.9) | <0.001 | 5(16.1) | 52(29.7) | 46(31.3) | 0.221 |
| 24≤BMI<27, N(%) | 70(20.6) | | 217(30.4) | 133(33.4) |  | 5(16.1) | 42(24.0) | 35(23.8) |  |
| BMI≥27, N(%) | 228(67.1) | | 397(55.6) | 170(42.7) |  | 21(67.7) | 81(46.3) | 66(44.9) |  |
| ESS, median(IQR) | 7(4-10) ^+^ | | 6(4-9) | 6(3-8) | .003 | 7(4-9) | 7(4-9) | 5(3-9) | .166 |
| ESS>10, N(%) | 64(18.8) | | 120(16.8) | 59(14.8) | .348 | 3(9.7) | 28(16.0) | 19(12.9) | .553 |
| AHI, median(IQR) | **32.1(14.8-63.8)** | | **30.6(14.9-54.8)** | **30.8(15.4-53)** | **.190** | **15.1(8.9-43.2)** | **14.3(8.7-29.6)** | **17.8(10.2-36.1)** | **.083** |
| mild, N(%) | 86(25.3) | | 182(25.5) | 96(24.1) | .965 | 15(48.4) | 93(53.1) | 63(42.9) | .398 |
| moderate, N(%) | 77(22.6) | | 170(23.8) | 98(24.6) |  | 6(19.4) | 40(22.9) | 39(26.5) |  |
| severe, N(%) | 177(52.1) | | 362(50.7) | 204(51.3) |  | 10(32.3) | 42(24) | 45(30.6) |  |
| RERA index, median(IQR) | 8.6(4.4-14.1) | | 8.7(4.9-15.1) | 9.1(4.5-15.9) | .629 | 8.2(3.7-17.6) | 8.6(4.1-13.8) | 8.2(3.5-15.1) | .910 |
| Sleep efficiency, %, median(IQR) | 85.5(72.7-91.9) ^* +^ | | 82.3(72.6-87.9)^#^ | 73.4(62.3-83.4) | .000 | 80.1(68.4-87) | 81.4(71.2-88.7) | 78(65.7-85.6)^#^ | .044 |
| Arousal index, median(IQR) | **16.3(10.6-28.8) ^+^** | | **15.6(9.6-25.8)** | **15.2(9.1-22.6)** | **.005** | **13.8(9.6-21.6) ^* +^** | **10.2(5.9-15.6)** | **9.9(5.1-15.7)** | **.014** |
| Oxygen desaturation index | 30.1(12.9-59.5) ^+^ | | 26.7(12.8-51.7)^#^ | 26.7(11.7-47.9) | .086 | 15.2(8.9-42.5) | 13.1(7.4-24.7) | 16.3(8.3-35.3) | .132 |
| SpO_2_ nadir, %, median(IQR) | 79(70-84) ^+^ | | 80(72-85)^#^ | 81(75-86) | .000 | 84(74-87) | 81(77-86) | 81(76-85) | .389 |
| SpO_2_< 90%, sec, median(IQR) | 12(2.6-50.1) ^+^ | | 10.1(2.2-36.6) | 8.5(2.1-29.6) | .043 | 2.1(0.5-9.6) | 4.1(1.1-13.8) | 5.8(1.5-20.4) | .123 |
| SpO_2_< 85%, sec, median(IQR) | 1.6(0.1-13.1) ^+^ | | 1.3(0-9)^#^ | 0.6(0-5.8) | .000 | 0.1(0-2.4) | 0.4(0-2.6) | 0.5(0-2.9) | .428 |
| SpO_2_< 80%, sec, median(IQR) | 0.1(0-2.6) ^+^ | | 0(0-1.9)^#^ | 0(0-0.9) | .001 | 0(0-0.5) | 0(0-0.4) | 0(0-0.4) | .873 |
| Chief complaint |  | |  |  |  |  |  |  |  |
| Snoring | 322(94.7) ^* +^ | | 624(87.4)^#^ | 326(81.9) | <0.001 | 28(90.3) | 163(93.1) | 129(87.8) | 0.254 |
| Daytime sleepiness | 307(90.3) ^* +^ | | 574(80.4)^#^ | 287(72.1) | <0.001 | 26(83.9) | 148(84.6) | 122(83.0) | 0.929 |
| Dozing off while watching TV | 181(53.2) | | 407(57.0) | 234(58.8) | 0.302 | 10(32.3) ^+^ | 95(54.3) | 96(65.3) | 0.002 |
| Dozing off while driving | 138(40.6)^+^ | | 310(43.4) | 79(19.8) | <0.001 | 2(6.5) | 40(22.9)^#^ | 8(5.4) | <0.001 |
| Habits |  | |  |  |  |  |  |  |  |
| Smoking | 124(36.5) | | 283(39.6)^#^ | 119(29.9) | 0.005 | 7(22.6) ^+^ | 15(8.6)^#^ | 2(1.4) | <0.001 |
| Drinking | 223(65.6) ^* +^ | | 406(56.9) | 205(51.5) | 0.001 | 15(48.4) ^+^ | 52(29.7)^#^ | 23(15.6) |  |
| Comorbidities |  | |  |  |  |  |  |  |  |
| Hypertension, N(%) | 74(21.8) ^* +^ | | 273(38.2)^#^ | 212(53.3) | <0.001 | 7(22.6) ^+^ | 57(32.6)^#^ | 84(57.1) | <0.001 |
| Heart disease, N(%) | 7(2.1) ^* +^ | | 67(9.4)^#^ | 97(24.4) | <0.001 | 1(3.2) ^+^ | 12(6.9)^#^ | 37(25.2) | <0.001 |
| Stroke, N(%) | 3(0.9) ^+^ | | 22(3.1)^#^ | 33(8.3) | <0.001 | 0(0) | 6(3.4) | 13(8.8) | 0.038 |
| Diabetes mellitus, N(%) | 16(4.7) ^+^ | | 78(10.9)^#^ | 77(19.3) | <0.001 | 4(12.9) | 20(11.4) | 29(19.7) | 0.109 |
| Hyperlipidemia, N(%) | 69(20.3) ^* +^ | | 267(37.4) | 120(30.2) | <0.001 | 7(22.6) | 70(40.0) | 69(46.9) | 0.038 |
| Anxiety, N(%) | 39(11.5) | | 90(12.6) | 71(17.8) | 0.019 | 6(19.4) | 40(22.9) | 44(29.9) | 0.249 |
| Depression, N(%) | 12(3.5) | | 43(6.0) | 28(7.0) | 0.109 | 5(16.1) | 23(13.1) | 28(19.0) | 0.352 |

BMI= body mass index. CSA=central sleep apnea. ESS=Epworth sleepiness score. IQR=interquartile range. OSA=obstructive sleep apnea.

^*^ post-hoc p<0.017 young adults(20-40 years) v.s. middle age adults (40-60 years),

^+^ post-hoc p<0.017 young adults v.s. older adults(>60 years),

^#^ post-hoc p<0.017 middle age adults v.s. elder adults

Table S2 Uni-variate logistic regression for odds ratio of characteristics related prevalence changes of OSA according to age

|  | Male |  |  | Female |  |  |
| --- | --- | --- | --- | --- | --- | --- |
|  | 20~40 year-old | 41~60 year-old | >60 years old | 20~40 year-old | 41~60 year-old | >60 years old |
| Smoking | 1.56(0.97-2.48)­ | 0.9(0.61-1.32) | 0.87(0.52-1.47) | 2.07(0.69-6.19) | 1.49(0.59-3.76) | 0.18(0.03-1.03) |
| Drinking | 1.27(0.82-1.96) | 1.03(0.7-1.52) | 1.01(0.62-1.65) | 1.34(0.58-3.13) | 0.66(0.4-1.08) | 0.87(0.39-1.97) |
| Obesity | **7.45(2.57-21.61)**** | **3.45(2.11-5.63)**** | 1.6(0.85-3) | **6.97(4.28-11.36)**** | **5.13(3.4-7.76)**** | **2.87(1.73-4.76)**** |
| Hypertension | **4.29(1.92-9.62)**** | **1.76(1.15-2.71)**** | 1.27(0.78-2.07) | **3.26(1-10.66)^+^** | **1.8(1.04-3.09)*** | 1.71(0.92-3.16) |
| Heart disease | 2.4(0.29-19.69) | 0.69(0.39-1.24) | 0.7(0.41-1.19) | 1.18(0.1-13.55) | 1.66(0.57-4.85) | 2.06(0.89-4.75) |
| Stroke | ^$^ | 0.75(0.28-2.02) | 0.89(0.38-2.1) | ^$^ | 4.15(0.49-34.96) | 1.29(0.4-4.12) |
| Diabetes mellitus | ^$^ | 1.25(0.64-2.42) | 1.06(0.57-2) | **10.67(1.14-99.75)*** | 1.56(0.69-3.56) | 1.76(0.72-4.27) |
| Hyperlipidemia | 1.47(0.82-2.62) | 1.07(0.72-1.6) | 1.06(0.62-1.82) | **6.81(1.63-28.43)**** | **2.91(1.67-5.06)**** | 1.22(0.66-2.26) |
| Anxiety | 1.23(0.61-2.48) | **0.59(0.36-0.98)*** | 0.57(0.32-1) | 0.79(0.28-2.24) | 0.67(0.4-1.14) | 1.45(0.71-2.95) |
| Depression | 0.66(0.24-1.81) | 0.92(0.42-2.01) | 0.5(0.23-1.08) ^+^ | 1.21(0.38-3.89) | 0.79(0.41-1.52) | 2(0.78-5.12) |

$:not available, ^+^:p<0.10, *: p<0.05, **: p<0.01

Figure S1


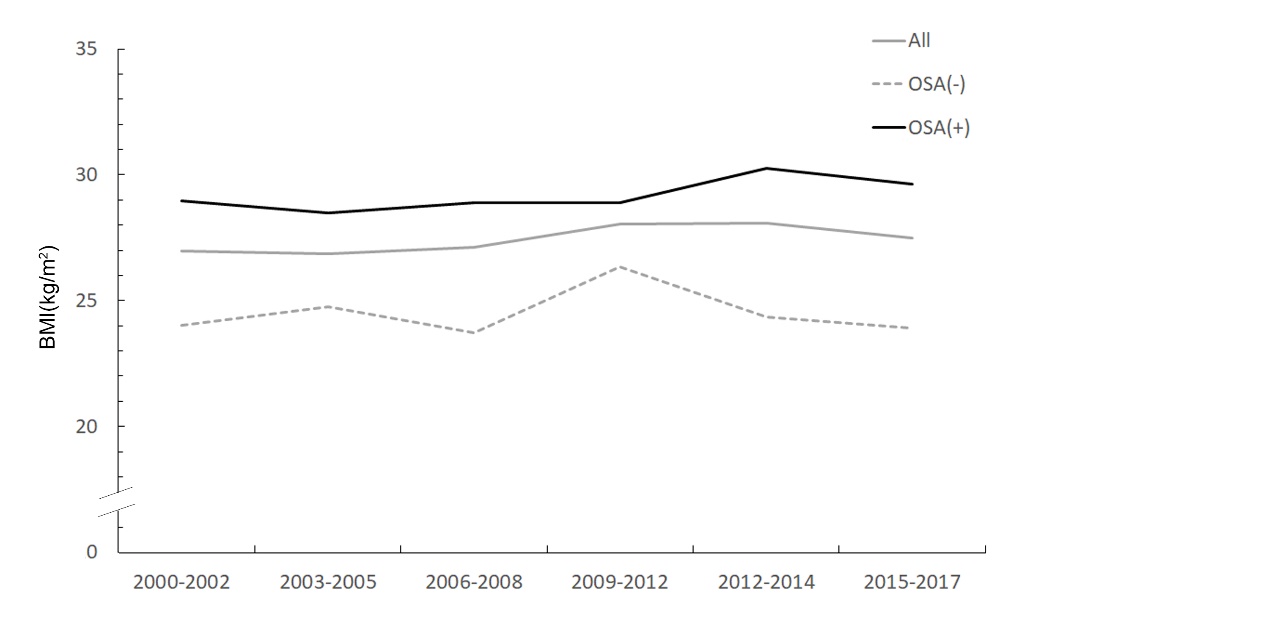


Title:

The trend of BMI in all young adults received PSG and young adults diagnosed with or without OSA.

Captions:

Though the trend of BMI of all PSG examinees remained statistically the same, the BMI of young adults diagnosed with OSA increased.

p value for trend: All:0.072, OSA(+):0.045, OSA(-):0.680

BMI=body mass index, PSG=polysomnography, OSA=obstructive sleep apnea.
